# Current approaches to studying membrane organization

**DOI:** 10.12688/f1000research.6868.1

**Published:** 2015-11-30

**Authors:** Thomas S. van Zanten, Satyajit Mayor

**Affiliations:** 1 National Centre for Biological Sciences (TIFR), Bellary Road, Bangalore, 560065, India

**Keywords:** membrane, electron microscopy, proteins

## Abstract

The local structure and composition of the outer membrane of an animal cell are important factors in the control of many membrane processes and mechanisms. These include signaling, sorting, and exo- and endocytic processes that are occurring all the time in a living cell. Paradoxically, not only are the local structure and composition of the membrane matters of much debate and discussion, the mechanisms that govern its genesis remain highly controversial. Here, we discuss a swathe of new technological advances that may be applied to understand the local structure and composition of the membrane of a living cell from the molecular scale to the scale of the whole membrane.

## Introduction

“The stone age did not end because we ran out of stones” ^1^ …

There has always been a close association between technological advancement and new research questions. A more recent example is how the application of x-ray crystallography to studying protein structures has opened up the possibility to elucidate structure and relate it to function ^2– 4^. In particular, how can single molecules transfer the genetic code into chemical and structural information? Research on the structure and organization of the cell membrane is undergoing a similar revolution with the application of (nano-)technological tools for the observation of membrane structure and composition.

**Figure 1.**
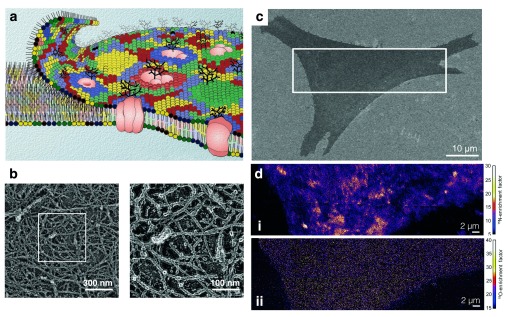
A chemical view of the cell membrane. ( **a**) Image of membrane bilayer exhibits a patchwork mosaic of the distribution of lipids in the cell membrane and captures the lateral heterogeneity of the organization of membrane components in live cells. ( **b**) This bilayer is anchored to the cortical actin meshwork as visualized by rapid-freeze deep-etch tomographic renderings of the cortical surface closest to the membrane. ( **c**) Scanning electron microscopy image of a fibroblast cell. ( **d, i**) The distribution of metabolically incorporated 15N-sphingolipids in the plasma membrane region indicated above represented as the detected sphingolipid-specific 15N-enrichment with NanoSIMS. Orange and yellow regions represent plasma membrane domains that are enriched with 15N-sphingolipids. ( **d, ii**) The distribution of 18O-enrichment showing that the metabolically incorporated 18O-cholesterol is distributed relatively uniformly in the plasma membrane. Images ( **a**), ( **b**), and ( **c**) and ( **d**) are reproduced with permission from 6, 19, and 35, respectively.

The outer membrane of the living cell is the interface that demarcates the cell and its environment. Communication in either direction takes place largely via the local arrangement of proteins and lipids at the plasma membrane. Decades of research on the mobility and spatial organization of components in the membrane by fluorescence microscopy and electron microscopy (EM), respectively, have suggested that the membrane is structured as a fluid lipid bilayer ^5^. More recent studies indicate that the membrane of the cell is not a simple fluid where lipids form a well-behaved two-dimensional (2D) fluid and where proteins are solutes in this milieu. Instead, the plasma membrane is organized as a dynamic mosaic whose local assemblies can span nano- to mesoscopic scales ^6^ ( Figure 1a).

It is important to understand how this organization, whether caused by thermodynamic fluctuations ^7^ or driven actively ^8, 9^, arises since it plays a significant role in the functioning of molecules embedded in this matrix. To build up a mechanistic understanding of how the cell effects this membrane organization, it is vital to have chemical, spatial, and temporal information of components in the cell membrane. Several technological advances are beginning to address these fundamental questions in more detail, and we will highlight how these are leading to a more complete picture of the cell membrane.

## High-resolution structural imaging

At the highest resolution, EM offers an unprecedented opportunity. With the development of cryo-EM tomography and of new detectors ^10^, the possibilities of imaging molecular organization inside the cell are unparalleled. Macromolecular protein complexes of interest can be seen in the context of their natural environment with resolutions beyond the nanometer scale ^11– 13^. Contrast in EM, however, is chiefly dependent on electron density and has been directed to inquiries involving defined structures such as the cytoskeletal ^14, 15^, endocytic cups ^16, 17^, and adhesion plaques ^13, 18^. Noteworthy are several reports visualizing the interaction of the cortical actin with the membrane ^11, 19^ where actin meshwork-like structures ( Figure 1b), aside from providing mechanical stability and shape, could impede membrane protein diffusion ^19^. The size and dynamics of these structures might well prove to be important for membrane-related reactions, priming specific cell function ^20, 21^. EM becomes especially powerful when combined with chemical specificity in the form of genetically tagged contrast agents ^22^ or through combination with fluorescence localization techniques ^23– 25^. However, obtaining chemically precise information with high resolution remains challenging.

## Chemically parsed spatial localization

The plasma membrane of any animal cell is composed of over 1,000 different types of lipids and proteins, presumably each with a specific purpose. Together, this assortment of chemical species at the plasma membrane primes the cell to adjust and react to the external milieu and communicate information about its internal state. Composition of both protein and lipid of the plasma membrane changes dramatically, depending on cell type ^26^, developmental stage ^27^, and pathological state ^28, 29^.

With the advent of sensitive mass spectrometry, it is now possible to construct a quantitative map of the entire protein and lipid composition of biochemically purified membranes ^30, 31^. The resulting *lipidome* or *proteome* of the membrane is evidence of the different sets of molecules that work together in space and time to perform function and process information. Access to their localization can be achieved (a) by label-free methods and (b) via high-contrast imaging of individual species.

### Label-free localization

Armed with a complete chemical composition of the membrane, obtaining the spatial organization of this information, preferably in real time and in a live cell, is the important next step. Matrix-assisted laser desorption ionization (MALDI) can locally vaporize material into ionized molecules or molecular fragments which are subsequently analyzed with a mass spectrometer. Raster-scanning the laser over a sample will generate an image with unprecedented chemical resolution, albeit at the rather low spatial scale of a few micrometers ^32^.

At the expense of chemical bandwidth, magnetic sector secondary ion mass spectrometry (NanoSIMS) offers a typical spatial resolution of 100 nm on cell membranes ^33^. In this method, a focused primary ion beam sputters neutral and ionized molecular fragments from the sample surface. These ejected secondary ions are subsequently collected and analyzed in the mass spectrometer. An additional benefit is a shallow sampling depth of 5 nm, which is due to the small secondary ion escape depth, making this technique exquisitely sensitive to the plasma membrane ^34^. Because of the monoatomic and diatomic nature of the secondary ion component, identification is possible only if the molecules of interest contain distinct elements or isotopes ^33^. For different lipids, this can be achieved by culturing cells in the presence of isotope-labeled precursors leading to their metabolic incorporation into the lipid of interest, which would have the same chemical structure as its unlabeled analogue.

Chemical mapping of the plasma membrane displayed 200 nm domains showing a significant sphingolipid enrichment ^34^ ( Figure 1c,d). These domains were further non-randomly assembled in patched regions that were about 3–10 μm in size ^34, 35^. Simultaneous chemical imaging of isotope-labeled cholesterol revealed that cholesterol, in contrast to sphingolipids, distributed in an apparent homogeneous fashion on the dorsal/upper membrane ^35^ analogously to a recent dynamic study ^36^. Despite the non-overlapping spatial distribution, cholesterol depletion did disperse the sphingolipid-enriched domains. Actin depolymerization had a more dramatic effect, suggesting a link between lipid organization and the actin architecture ^35, 37^. With the possibility to include specific protein labeling along with the mapping of lipid components at the nanometer scale, this technique will continue to contribute to our understanding of the spatial distribution of chemistry in the membrane ^38, 39^.

The benefit of label-free methods is avoiding the possible influence of an attached label on the behavior of the specific protein or lipid of interest; for any lipid, given the mass ratio of a fluorescent label to the mass of the lipid species, this perturbation is likely to be substantial. Nevertheless, to get more detailed knowledge of how a cell constructs complexes in a membrane, molecular recognition with a high signal-to-noise ratio in an aqueous scattering milieu, a feature that label-free methods still lack, is essential.

### High-contrast imaging

Increase of signal-to-noise ratio is attained by specifically targeting or labeling the molecule of interest with, for example, an antibody, genetically, or by chemically incorporating a contrast agent. If the labels are carrying electron-dense material ^22^ or conjugated with gold nanoparticles ^40^, they can be visualized with an electron microscope. In general, however, fluorescence light microscopy is used where individual targets of interest are coupled to fluorophores.

Focusing of light, however, is inherently diffraction-limited. With lens-based optics, light cannot be focused better than about 200–300 nm and individual objects that are spaced closer cannot be distinguished as unique objects anymore. To overcome this concentration limit ^41^, several approaches have come up in the last decade to either (temporally) dilute the observed molecules (stochastic super-resolution microscopy ^42, 43^) or decrease the observation volume (targeted super-resolution microscopy ^44, 45^). Single-molecule imaging ^46^ has opened up a major avenue not only to observe the localization of single fluorophores at very high spatial resolution but also to study the biochemistry of individual species to derive ensemble properties of molecules inside a cell.

### Photo-localization microscopy

Optically interrogating the dynamic behavior of single molecules in their highly concentrated presence on the plasma membrane is made feasible by isolating a fluorescently labeled representative. Although the membrane components still move in their natural environment, their dynamics can now be characterized by recording the motion of a number of such ‘single representatives’ on a camera. If the distance between the individual molecules in each image is larger than the diffraction limit, their positions can be determined with nanometer precision ^47, 48^. This accuracy of determining its center-of-mass is essentially inversely proportional to the square root of the number of photons emitted ^47^. The positions of multiple fluorescent spots can be identified and related to their position in earlier images to build up their time trajectories ^49^. The number of molecules can be tuned via the concentration of externally added specific markers, photo-activation of only a subset of fluorescent molecules ^50– 52^, or the photo-bleaching of a well-defined area followed by the sparse diffusion back in the observation area ^53, 54^. At the other end, technical advances in hyper-spectral detection should increase single-particle discrimination allowing an increase in concentrations of single molecular representatives ^55^.

**Figure 2.**
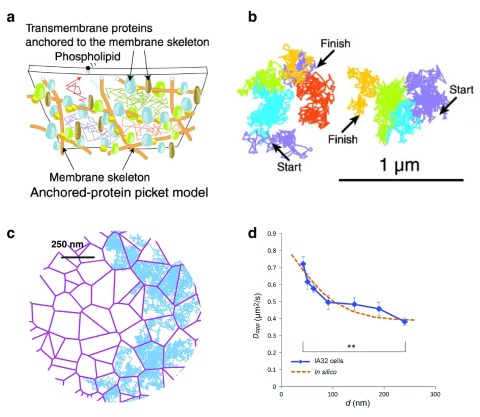
A dynamic view of the membrane. ( **a**) Picket-fence model where transmembrane proteins anchored to the actin membrane skeleton meshwork effectively act as rows of pickets and temporarily confine the movement of lipids and proteins through steric hindrance and circumferential slowing (packing or frictional) effects. ( **b**) Two representative trajectories of 1,2-dioleoyl- *sn*-glycero-3-phosphoethanolamine (DOPE) lipids on a living cell membrane recorded at a time resolution of 25 μs (40,500 frames/s) for a period of 56 ms (2,250 frames) where plausible compartments are shown in different colors. ( **c**) Schematic Voronoi lattices (purple) representative of actin-based compartment sizes together with simulated diffusion trajectories (cyan). Scale bar: 250 nm. ( **d**) Dependency of the apparent diffusion coefficient of di-palmitoyl phosphoethanolamine (DPPE) lipids on the area of observation (blue line). Images reproduced with permission from 69 ( **a**, **b**) and 135 ( **c**, **d**).

Recording a sufficient number of tracks or a single molecule for a sufficiently long time builds up the statistical behavior of the membrane components in terms of the diffusion coefficient or type of mobility ^56^. Individual trajectories pooled into a distribution of diffusion coefficients can then be related to the functional/affinity state of a receptor ^57, 58^. Local changes in the individual trajectory can be mapped out on the cell to indicate the nature of the area traversed ^59, 60^, in terms of diffusion ^50^, confinement regions ^61^, or local energetic changes ^62^. Examining the individual tracks of receptors as they diffuse in the plasma membrane revealed that they could become obstructed by lipid domains ^63^, protein-protein interaction ^64^, tetraspanin network ^65^, glycan structures ^59, 66^, or the actin cytoskeleton ^67– 69^ ( Figure 2a,b).

Detection of changes due to interactions or confinement within boundaries of a compartment is reflected in changes in the molecular diffusion coefficient, which in turn depends on interaction strengths, acquisition speed, and signal-to-noise ratio (localization accuracy) ^70^. On the other hand, prior information on molecular mobility could facilitate teasing out a subset of molecules without effectively diluting the experiment. If the subset (of interest) moves significantly slower because of an activation event or substrate binding, it is possible to experimentally deconvolve out the contribution of individual players in the reduction of mobility ^60, 71^. Using relatively long exposure/integration times, fast-moving fluorescent molecules blur into the background while slower moving molecules emit photons from the same diffraction-limited volume and therefore can be localized.

### Stochastic super-resolution microscopy

The stochastic cycling of dyes between fluorescent *on*-states and non-fluorescent *off*-states is an unfavorable property for single-molecule tracking because the single molecule might become undetectable in several frames and get lost during the trace reconstruction. Alternatively, if tuned properly, this cycling between states can be used to temporarily dilute highly concentrated samples. The challenge is to have at each given time only a subset of molecules in the *on*-state and determining the center-of-mass for each molecule before they switch *off* again. If this process is repeated many times, all of the calculated positions can be used to reconstruct a “super-resolution” image ^72^. This indeed is the concept of the localization techniques called stochastic optical reconstruction microscopy (STORM) ^73^ and (fluorescent) photoactivatable localization microscopy, or (f)PALM ^42, 43^. Whereas STORM is essentially based on organic dyes that reversibly switch between *on*- and *off*-states ^74– 77^, PALM is based on engineered fluorescent proteins ^78– 80^.

**Figure 3.**
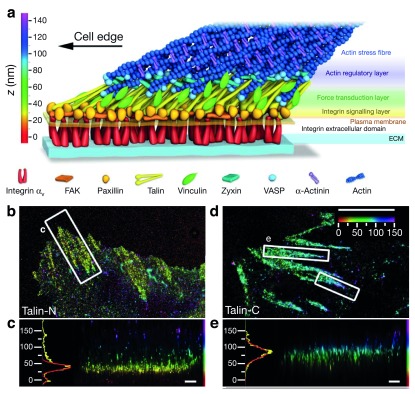
A super-resolution view of membrane associated focal adhesions. ( **a**) Schematic model of the molecular architecture of focal adhesions. This model is based on the protein position measurement by interferometric photoactivatable localization microscopy (iPALM). The exquisite sensitivity of iPALM to register axial distances could determine the orientation of talin within focal adhesion. ( **b**– **e**) Top and side views of iPALM images of focal adhesions (white boxes, top-view panels) and corresponding z histograms. Color encodes distance from the coverglass surface in nanometers. Placing the fluorescent probe at the two ends of the talin rod show that the N-terminus of talin rod localizes close to the cytoplasmic tails of integrin ( **b**, **c**), whereas the C-terminus can localize up to 40 nm higher ( **d**, **e**). Scale bars: 5 μm ( **b**, **d**) and 500 nm ( **c**, **e**). Images reproduced with permission from 89. Abbreviations: ECM, extracellular matrix.

Photo-localization-based super-resolution is excellent in determining sub-resolution complexes such as the endocytic clathrin-coated pits ^81, 82^, microtubular structures ^83– 85^, cytoskeletal structures ^86, 87^, and adhesion plaques ^88, 89^ ( Figure 3). Quantitative analysis of super-resolved domains in the plasma membrane in terms of absolute number of molecules is challenging because of blinking ^90^ and activation efficiency ^91^. Nevertheless, analytical methods such the pair-correlation ^92^ and Ripley’s K function ^93, 94^ have allowed a certain degree of quantification, but this is strongly dependent on the assumptions employed in applying these statistical analyses to the data.

Since photo-localization-based super-resolution is based on a random spatial sampling of the structure, activating or switching light pulses are not necessarily required. In fact, it is possible to make use of the intrinsic trait of fluorophores to get temporarily trapped in a dark state, blinking. By engineering fluorophores that have longer dark states ^95^ or exploiting the known on-off blinking of quantum dots ^96^, the chances that nearby emitters are both in an *on*-state decrease. On the other hand, one could tune incorporation rates of fluorescent species to the membrane (proteins); in a bath of freely diffusing fluorescent ligands, only the temporarily bound ligands will be detected, essentially taking advantage of mobility difference between bound and unbound ^97– 99^.

### Targeted super-resolution microscopy

Super-resolution imaging in the context of breaking Abbe’s diffraction limit has been achieved by decreasing the observation volume below the diffraction limit of light. This has been mainly accomplished with (a) use of near-field geometries or restricted physical apertures to confine the excitation volume or (b) the clever use of lasers to selectively deplete excited fluorophores in all except the very center of the optical volume to confine the emission volume.

***(a) Near-field optics.*** By physically confining the light inside a very small aperture of 50–150 nm in diameter, light propagation is discontinued and the electromagnetic fields become restricted to the aperture (i.e., to the near field). The light intensity exponentially decays away from the aperture, producing essentially a nanoscopic excitation source. For imaging purposes, such a sub-wavelength aperture is created in a tapered aluminum-coated optical fiber. The image can then be built up by raster-scanning the aperture in close proximity to the sample and in fact this was one of the first approaches to obtain super-resolution images ^45^. Because the rendering of the image is independent of the photo-physical properties of the sample/fluorophore, it allows near-field scanning optical microscopy (NSOM) to obtain quantitative information at the nanometer scale. Additionally, the same physical aperture confines multiple wavelengths and the technique is therefore free from chromatic aberrations ^100, 101^. Colocalization ^102^ among multiple chemical species (due to biochemical interaction), random scattering ^101^ (due to the lack of interaction), and segregation ^103^ are not the only modes of organization. Indeed, multicolor super-resolution imaging revealed multi-domain proximity on the order of 50–150 nm as another tendency ^101, 104, 105^. Diffraction-limited techniques would erroneously identify such proximity as colocalization, showing the merit of any super-resolution technique. Proximity does not preclude interaction, and quantitative analysis showed that integrin activation could bias the glycosyl phosphatidylinositol-anchored protein (GPI-AP) organization to a more clustered state ^105^. This more detailed quantification was granted by the possibility of having single-molecule sensitivity at the nanometer scale. Practical resolution of NSOM, however, is limited to approximately 50–70 nm, driving the field toward the use of optical antennas. Optical antennas, analogous to their radio frequency equivalent, convert freely propagating electromagnetic radiation into localized energy, and vice versa. Initial experiments have shown resolutions of 30–50 nm of proteins on a plasma membrane using a gold nano-particle ^106^ or a sculpted monopole ^107^ as photonic antenna. Recent advances in antenna design provided simultaneous multicolor localization accuracies well below 1 nm with low photon budgets ^108^, showing tremendous potential for nanoscale sensing or imaging ^109^. Near-field imaging or spectroscopy, however, is confined to sample surfaces that are accessible to the physical aperture/probes, making *in vivo* imaging inside cells a very difficult proposition.

***(b) Stimulated emission depletion.*** A different strategy toward true super-resolution is to confine the emission instead of the excitation ^110^. Its principle is based on the positionally deterministic switching of the fluorophore state in contrast to the stochastic switching for localization-based super-resolution ^111, 112^. Stimulated emission depletion (STED) microscopy is founded on depleting the excited state of a fluorophore by stimulating the excited fluorophore to emit a photon of specified wavelength. By creating a highly intense donut-shaped emission depletion region around the confocal excitation volume, only the fluorophores in the center of the donut are spontaneously emitting in the detected wavelengths. By increasing the intensity of the depletion donut, the resolution of the microscope is increased. STED imaging has been used to identify and quantify cluster sizes in cell membranes ^59, 113– 115^. The cluster sizes ranged from 50 to 160 nm, and STED experiments on membrane sheets indicated that the protein clusters were fine-tuned by electrostatic interactions and that these clusters are further assembled in relatively stable multi-protein assemblies ^116^. Further development of the technique toward parallelization ^117, 118^, multicolor acquisition ^119– 121^, and different illumination schemes ^122, 123^ will definitely increase imaging capacity. Better spatial resolution, however, is accompanied by an increased *on*- *off* cycling load on the fluorophore during scanning, requiring further progress in fluorophore engineering.

### Fluorescence fluctuation spectroscopy

Instead of demanding the heavy burden of photo stability from the fluorophore during multiple rounds of irradiation in the course of image build-up, one could allow the molecule itself to diffuse through the observation volume. During this passage, a fluorescent molecule will cause an intensity burst that is inversely proportional to the number of molecules in the observation volume. When sufficient numbers of molecules have passed, the detected intensity fluctuations can be autocorrelated, designating the technique as fluorescence correlation spectroscopy (FCS). The time at which this autocorrelation function decays to half its original value corresponds to the characteristic timescale at which the molecules move through the observation volume.

There is a linear relationship between the diffusion time and the area of observation for 2D diffusion in the plasma membrane. The slope of this relation is inversely proportional to the apparent diffusion coefficient, and the time-axis intercept, obtained from extrapolation, is indicative of confinement ^124^. According to this methodology, particles diffusing in the membrane can be divided in three major categories: (a) random diffusive, (b) domain interacting, and (c) meshwork constrained ^125, 126^. Exploitation of the FCS diffusion-law methodology found that sphingolipid- and cholesterol-dependent nanoscale domains are crucial for signaling ^127^.

Similar methods of optically diluting the sample, as described above, can be employed for FCS ^128^. More powerful is the combination of super-resolution techniques that confine the observation volume with FCS since this allows the registration of dynamics at the nanometer scale ^129– 132^. Mobility characteristics at the nanometer scale do not have to be extrapolated anymore ^125, 126, 133^ but can be directly measured ^130, 134, 135^ ( Figure 2c,d). In fact, extensive research using the tunable nanoscopic observation volume provided by STED indicated that fast-moving lipid analogues exhibit distinct modes of mobility that can be divided in three classes ^130, 136– 139^: (a) weak interactions of phosphoglycerolipids, (b) cholesterol-assisted binding mediated by the ceramide group, and (c) hydroxyl headgroup-assisted cholesterol-independent binding. In the future, bridging length scales with, for example, camera-based FCS ^140– 142^ or spatio-temporal image correlation spectroscopy ^143– 146^ should allow the visualization of how these fluctuating nanoscale assemblies can be stabilized to coalesce into functional signaling platforms ^7^.

With the advent of reproducible nanofabrication techniques, a whole new field lies open for exploration. Engineered substrates can provide aperture-based ^134, 147– 150^ or optical antenna-based ^151, 152^ nanofocusing of light on conventional microscopes. By virtue of the cells adhering to the substrate, the plasma membrane is brought in the near field of various nanoscopic excitation sources. Each of these excitation hotspots can now be addressed to locally probe membrane dynamics down to 20 nm ^152^ or in a multicolor fashion ^150^.

## Measuring molecular interactions in the membrane

Multiple components in the cell membrane work together and interact to effectuate signaling. Interacting molecules would diffuse together through the excitation volume. Measuring the fluorescence cross-correlation in an FCS setup will therefore display a correlation proportional to the interaction between the two particles ^153, 154^. Cross-correlation analysis among probes with different membrane anchoring units suggested domain formation but reiterated the notion of a complex underlying machinery ^155^ that is not necessarily instructed by phase transitions ^156^.

**Figure 4.**
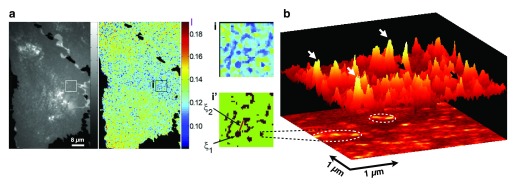
A spatial view of the cell membrane: hierarchical organization of proteins. ( **a**) Quantitative analysis of the spatial distribution of glycosyl phosphatidylinositol-anchored proteins (GPI-APs). On flat regions of relatively constant fluorescence intensity (grayscale), the anisotropy images (pseudocolored, where low values indicate increased numbers of clusters) display a hierarchical distribution of GPI-AP (e.g., in the form of nanoclusters and characteristic distances between nanocluster-rich regions). *Homo*-Förster resonance energy transfer ( *h*-FRET) imaging reports on the molecular proximity of like fluorophores at the 1- to 10-nm scale but imaging is still diffraction-limited and has no access to the region between 10 and 300 nm. ( **b**) A super-resolution technique such as near-field scanning optical microscopy (NSOM) can provide access to these spatial scales and revealed GPI-AP nanoclusters to organize in 150- to 300-nm sized regions. Three-dimensional projection of a fluorescence intensity NSOM image with nanodomains (black arrows) and monomers (white arrows) of GPI-APs is shown. Contour dashed lines on the two-dimensional image illustrate the preference of nanodomains to concentrate on specific sites as hotspots. Images reproduced with permission from 165 ( **a**) and 105 ( **b**).

As an alternative to multi-particle tracking ^157^ and cross-correlation spectroscopy ^158^, a more direct path to uncover nanoscale multi-molecular mixing is Förster resonance energy transfer (FRET). Here, the energy of the excited state of a fluorophore (donor) is non-radiatively transferred to a neighboring fluorophore (acceptor). Upon returning to the ground state, the acceptor fluorophore subsequently emits a photon with different characteristics—lifetime ^159^, polarization ^160^, or Stokes-shifted ^161^—as compared with an unperturbed donor fluorophore. Measurement of energy transfer between like fluorophores, *homo*-FRET, has been instrumental in the determination of small actively maintained nanoclusters ^8, 162^ ( Figure 4a). The constant fraction of dense nanoclusters at a large range of concentrations ^163– 165^ together with large fluctuations in local density distribution ^165, 166^ of lipid-anchored proteins is inconsistent with thermal equilibrium. Recognizing that a cortical layer of actin and myosins can drive membrane components by the consumption of energy is reconcilable with non-equilibrium membrane organization. The resolution of the FRET signal is intrinsically limited by the optical resolution; however, combining super-resolution methods as those described above with FRET opens up using the information of molecular proximity at the nanometer scale with structure and organization at the tens of nanometers offered, for example, by NSOM ( Figure 4b).

In such a composite, intermolecular connections in the membrane are being complemented by the interactions of each component with the underlying cortical actin ^9^. Although *inert* particles are not influenced by the cortical actin ^165, 166^, interactions of *passive* particles to the actin can be either direct ^166^ or indirect ^167^. The formations of these local domains can subsequently be used as a signaling platform ^127, 157, 168^. At the same time, the acto-myosin-dependent localization of domain creation and dissipation allows the cell to tune information-processing capacities of *passive* particles ^20, 169^. In this context, one can define a third class of membrane components as *active*: the membrane particles preoccupied with the tuning process. Examples of this last category are integrins ^170, 171^, G protein-coupled receptors ^172^, and T-cell receptors ^173, 174^, all of which are intricately involved in cortical actin reshaping.

## Outlook on disentangling molecular function

Precise positional information of proteins, even in the context of their chemical and topographical environment, might not always be enough to tease out how a particular mechanism works. New tools such as sensors that can identify activation state will help elucidating the spatial patterns behind activating signals ^175– 178^. On the other hand, one could effectively influence the system via substrate-controlled calibration ^179^, recruitment ^180, 181^, or perturbations ^182, 183^ and optogenetic regulation of protein (de)activation ^184– 187^. A completely different but nevertheless informative approach would be an *in vitro* assay to help untangle a mechanistic understanding of cellular behavior ^188– 193^. By taking the process out of the cell, one can rebuild step-by-step and investigate the minimal chemistry required to regain function ^194^.

**Figure 5.**
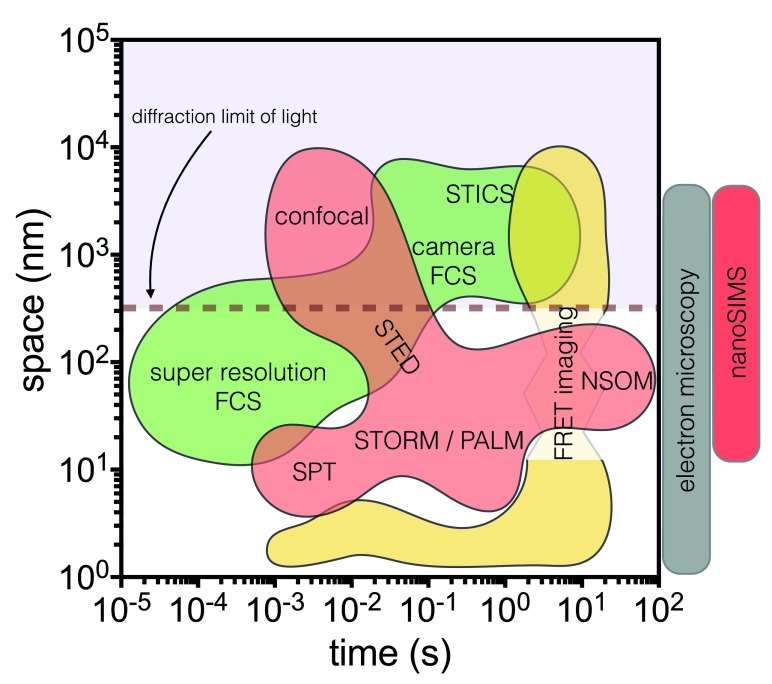
Space and time resolution of current methods. Representation of the landscape that current methods occupy in the space and time axes. Abbreviations: FCS, fluorescence correlation spectroscopy; FRET, Förster resonance energy transfer; NSOM, near-field scanning optical microscopy; PALM, photoactivatable localization microscopy; SPT, single particle tracking; STED, stimulated emission depletion; STICS, spatio-temporal image correlation spectroscopy; STORM, stochastic optical reconstruction microscopy.

A growing myriad and fruitful blend of interdisciplinary methodologies and technical improvements are shedding light on the spatio-temporal fluctuations of functional chemistry that underlies cellular behavior. This is captured in a 2D plot that maps the landscape of these possibilities ( Figure 5). Of note is the observation that a large fraction of the area of this map is occupied, and only a few regions remain unpopulated by methods available today. It is only a matter of time until we can directly follow the evolution of nanoscale heterogeneities to microscale patterning of plasma membrane components after receptor activation.

## Abbreviations

2D, two-dimensional; EM, electron microscopy; FCS, fluorescence correlation spectroscopy; FRET, Förster resonance energy transfer; NSOM, near-field scanning optical microscopy; PALM, photoactivatable localization microscopy; STED, stimulated emission depletion; STORM, stochastic optical reconstruction microscopy.
